# Comparing recalled versus experienced symptoms of breathlessness ratings: An ecological assessment study using mobile phone technology

**DOI:** 10.1111/resp.14313

**Published:** 2022-06-13

**Authors:** Jacob Sandberg, Josefin Sundh, Peter Anderberg, David C. Currow, Miriam Johnson, Robert Lansing, Magnus Ekström

**Affiliations:** ^1^ Department of Clinical Sciences, Division of Respiratory Medicine & Allergology Lund University Lund Sweden; ^2^ Department of Respiratory Medicine, School of Medical Sciences Örebro University Örebro Sweden; ^3^ Department of Health Blekinge Institute of Technology Karlskrona Sweden; ^4^ Wolfson Palliative Care Research Centre, Hull York Medical School University of Hull Hull UK; ^5^ IMPACCT, Faculty of Science, Medicine and Health University of Wollongong Wollongong New South Wales Australia; ^6^ Department of Psychology University of Arizona Tucson Arizona USA

**Keywords:** breathlessness, dyspnoea symptoms, mEMA, mobile ecological momentary assessment, peak‐end rule, recall of symptoms

## Abstract

**Background and objective:**

Recall of breathlessness is important for clinical care but might differ from the experienced (momentary) symptoms. This study aimed to characterize the relationship between momentary breathlessness ratings and the recall of the experience. It is hypothesized that recall is influenced by the *peak* (worst) and *end* (most recent) ratings of momentary breathlessness (peak‐end rule).

**Methods:**

This study used mobile ecological momentary assessment (mEMA) for assessing breathlessness in daily life through an application installed on participants' mobile phones. Breathlessness ratings (0–10 numerical rating scale) were recorded throughout the day and recalled each night and at the end of the week. Analyses were performed using regular and mixed linear regression.

**Results:**

Eighty‐four people participated. Their mean age was 64.4 years, 60% were female and 98% had modified Medical Research Council (mMRC) ≥ 1. The mean number of momentary ratings of breathlessness provided was 7.7 ratings/participant/day. Recalled breathlessness was associated with the *mean*, *peak* and *end* values of the day. The *mean* was most closely associated with the daily recall. Associations were strong for weekly values: *peak breathlessness* (beta = 0.95, *r* ^2^ = 0.57); *mean* (beta = 0.91, *r* ^2^ = 0.53); and *end* (beta = 0.67, *r* ^2^ = 0.48); *p* < 0.001 for all. Multivariate analysis showed that *peak breathlessness* had the strongest influence on the breathlessness recalled at the end of the week.

**Conclusion:**

Over 1 week, recalled breathlessness is most strongly influenced by the *peak breathlessness*; over 1 day, it is *mean breathlessness* that participants most readily recalled.

## Graphical abstract

Recall of breathlessness is essential for clinical care but might differ from the momentary symptoms. This study reports that the peak momentary breathlessness most strongly influences recalled breathlessness over the past 7 days. Recall for 1 day was influenced the most by the *mean breathlessness value* for that day.

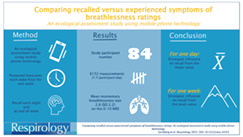

**See related** Editorial

## INTRODUCTION

Chronic breathlessness frequently affects the daily life of individuals with diseases such as congestive heart failure, asthma and chronic obstructive pulmonary disease (COPD).^1^ It is associated with increased use of health services, hospitalizations and premature mortality.^2^, ^3^, ^4^

In clinical practice, patient recall of recent breathlessness intensity is often used to assess the severity of conditions, establish the need for further examination and evaluate response to therapy. However, recall of symptom severity may not accurately reflect the patient's experiences across the time span in question.^5^, ^6^, ^7^, ^8^, ^9^, ^10^, ^11^ The process of reporting symptoms involves complex tasks, including recalling, summarizing and communicating past experiences.^6^, ^12^, ^13^ There is a wide variation in how patients approach this task, making interpretation of reported symptoms challenging.^12^, ^14^

The ‘peak‐end rule’ is related to a cognitive bias that influences the recall of past events.^6^, ^13^, ^15^ The rule states that the highest (peak) and most recent (end) intensity of a symptom during a specified time period has the most influence on the recalled symptom level. The peak‐end rule impacts the recall of a variety of situations such as painful procedures,^12^, ^13^, ^16^, ^17^, ^18^, ^19^ events evoking emotion,^20^, ^21^, ^22^, ^23^, ^24^ exercise^25^ and episodes of mental effort.^26^ However, the peak‐end rule seems to have a lower effect on the recall of more complex life experiences.^19^, ^27^, ^28^, ^29^ It is largely unknown which factors affect recall of breathlessness.^30^, ^31^, ^32^ Recall of breathlessness after exercise seems to differ from the recall of pain by being context‐dependent^30^ and less affected by the peak‐end rule.^5^, ^7^, ^9^, ^33^

A previous study using paper diaries showed that the intensity of breathlessness on the study day was the most important contributor to variations in recalled scores.^34^ More recently, mobile ecological momentary assessments (mEMA) for data collection have been shown to be both more reliable and lead to better compliance than paper diaries.^35^, ^36^, ^37^, ^38^, ^39^, ^40^

This study aimed to evaluate the relationship between recalled and experienced (momentary) ratings of breathlessness and determine whether the *mean*, *peak* or the *most recent* momentary rating has the strongest influence on the recall.

## METHODS

The Relating Experienced To Recalled Breathlessness Observational (RETRO) study is an observational study with longitudinal data collection for 1 week (7 days), using an application installed on participants' mobile phones for data collection (mEMA). A detailed description of all methods has been published.^41^ An mEMA STROBE checklist^38^ is in the supplementary material.

### Population and design

Inclusion criteria were age ≥ 18 years with a self‐reported breathlessness intensity ≥3 on a 0–10 numerical rating scale (NRS) during the preceding 14 days, not related to an acute infection such as an upper respiratory tract infection or pneumonia. Participants needed to be clinically stable, regularly use a smartphone or tablet with internet access and be able to read and complete baseline assessments on the device.

**FIGURE 1:**
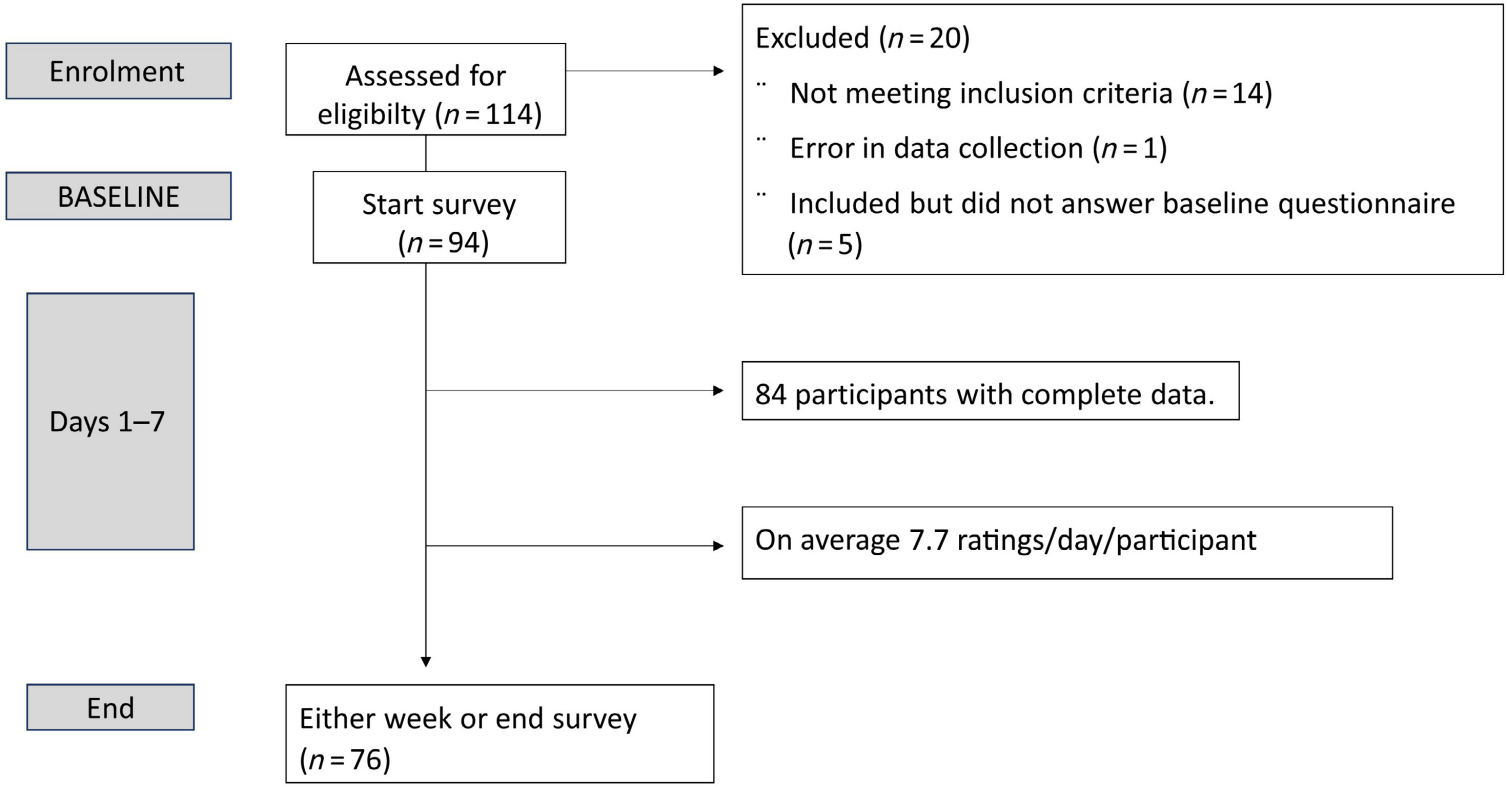
Study design and the inclusion of participants

From March 2018 to April 2020, participants were recruited via notice in a local newspaper; at primary care facilities in Lund and Karlskrona; at pulmonary clinics in Karlskrona and Örebro; and by invitation letter to patients of the Karlskrona pulmonary clinic. Potential participants installed an application on their personal smartphone and, if eligible, continued to a baseline questionnaire. All questionnaires were in Swedish. The participants were asked to respond to repeated questions each waking hour of the day as well as each morning and evening for 7 days. At the end of 7 days, or if choosing to drop out, participants were presented with an end of study questionnaire (Figure 1). No training of participants was needed.

### Assessment

Baseline characteristics included age, sex, height, weight, smoking habits and physician diagnoses. Breathlessness in the week before the study was assessed using the modified Medical Research Council (mMRC) breathlessness scale^42^ and a 0–10 NRS (‘How intense has your breathlessness been during the previous week’). The mMRC is a 5‐point ordinal measure of exertional breathlessness from 0 (‘I only get breathless with strenuous exercise’) to 4 (‘I am too breathless to leave the house’ or ‘I am breathless when dressing’). The mMRC responses 3 and 4 were merged due to a recording error. The NRS^43^, ^44^ is widely used and validated for the assessment of breathlessness.^45^, ^46^, ^47^, ^48^

Underlying conditions were reported by selecting them from a pre‐defined list. Momentary breathlessness was assessed using the question ‘How intense has your breathlessness been in the past 10–15 min?’, rated from 0 (no breathlessness) to 10 (worst imaginable breathlessness).^41^

Recalled breathlessness was assessed each evening using the NRS (‘How intense has your breathlessness been during this day?’) and at the end of the 7 days using the question ‘How intense has your breathlessness been during the past week?’.

### Statistical analysis

Outcomes were the *recalled breathlessness at the end of the day* and *recalled breathlessness for the week*. The exposure was the momentary breathlessness reported at times throughout the day. Momentary breathlessness was analysed as:

- The difference in breathlessness intensity between momentary ratings and the *mean* value for that day, assessing for impact on recall from a change in breathlessness;
- The *mean* of an individual's momentary breathlessness ratings for each day;
- The *mean* momentary breathlessness ratings for all of the 7 days;
- The *peak* value for each day defined as the highest reported momentary value of breathlessness;
- The *peak* value for the whole 7‐day study period;
- The last recorded (*end*) momentary breathlessness rating of each day; and
- The last recorded (*end*) momentary breathlessness rating of the entire study period.

Associations between momentary and recalled breathlessness ratings throughout the day were analysed using mixed linear regression with random intercepts and slopes, with clustering by participant. This model allows the intercept (mean level of momentary breathlessness) and the slope (change in momentary breathlessness) to vary among participants. Clustering accounted for repeated measurements within participants' responses during the analysis period.

Associations were reported as beta coefficients with 95% CIs. A beta coefficient is defined as the mean change in the outcome variable (the recalled value for the day) for each unit increase of the exposure value (the momentary breathlessness measures recorded during the day).

Associations over the 7 days were analysed using linear regression. The recalled breathlessness for the entire study period was the dependent variable, and *mean, peak* and *end* (last recorded) values of momentary breathlessness during the week were the independent variables. The variables were analysed separately and pairwise in multivariate analysis models 1–3 and combined in a final model. The variance inflation factor (VIF) was used to check for multi‐collinearity. Low VIF values were found, indicating that the risk of multi‐collinearity was low (highest VIF = 3.8).

Beta coefficients with 95% CI and the corresponding adjusted *r* ^2^ value (reflecting the percentage of the variance explained by the model) are presented. The unique contribution of each factor to each model was assessed by calculating the Δ*r* ^2^ for each factor by subtracting the variable's *r* ^2^ values from the *r* ^2^ value of the entire model. Significance was defined as two‐sided *p* < 0.05.

A power analysis performed before the enrolment began^41^ determined that a minimum of 30 participants was needed to obtain a power of 80%, consistent with the sample size of Meek et al.^34^ We aimed for at least 45 participants providing data for at least 2 days. The statistical analysis plan was designed in collaboration with a biostatistician.

Statistical analyses were conducted using the software package Stata, version 14.2 (StataCorp LP, College Station, TX).

## RESULTS

### Participants

A total of 114 people downloaded the application, of whom 30 were excluded from the analysis based on: not meeting the eligibility criteria (*n* = 14); not completing the baseline questionnaire (*n* = 5); a technical error with the mobile phone application (*n* = 1); and not responding to enough daily prompts or not providing recall information (*n* = 10). Excluded individuals who contributed baseline data did not differ substantially from those included in age, sex or breathlessness level. The final study population comprised 84 individuals. A total of 8121 prompts for momentary breathlessness rating were sent out to the 84 participants, and 6152 were answered within 1 h (a mean of 7.7 ratings/participant/day). The other 1969 prompts were tagged as missing (compliance rate of 75.8%). Seventy‐six individuals completed the whole data collection period, including the end‐of‐study assessment (Figure 1).

The mean age of the study population was 64.4 (SD 12.8); 60% were female; and the main underlying diagnoses were COPD (40%) and asthma (39%). A total of 30% of the participants had never smoked (Table 1). Breathlessness during the preceding week was reported on the mMRC scale by 98% (grade 1 [37%], grade 2 [26%] or grades 3 and 4 [35%]).

**TABLE 1:** Baseline characteristics of the 84 study participants experiencing daily breathlessness

Characteristic	Value (%)
*n*	84
Age, mean (SD)	64.4 (12.8)
Female, *n* (%)	50 (60)
BMI, mean (SD)	28.2 (5.4)
Smoking status, *n* (%)	
Never	25 (30)
Former	54 (64)
Occasionally	2 (2)
Regular daily smoking	3 (4)
Breathlessness past week (0–10 NRS), mean (SD)	5.2 (1.8)
mMRC past week, *n* (%)	
0	2 (2)
1	31 (37)
2	22 (26)
3–4	29 (35)
Asthma, *n* (%)	33 (39)
COPD, *n* (%)	34 (40)
Heart failure, *n* (%)	7 (8)
Atrial fibrillation, *n* (%)	9 (11)
Coronary heart disease, *n* (%)	7 (8)
Cancer, *n* (%)	11 (13)
Diabetes, *n* (%)	9 (11)
Hypertension, *n* (%)	33 (39)
Stroke, *n* (%)	2 (2)

*Note*: Data were self‐reported by participants.

Abbreviations: COPD, chronic obstructive pulmonary disease; mMRC, modified Medical Research Council; NRS, numerical rating scale.

### Breathlessness data

**FIGURE 2:**
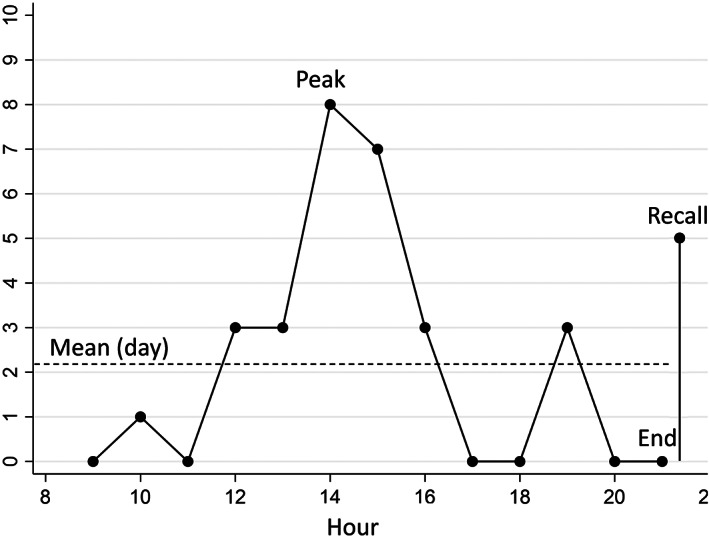
Momentary breathlessness ratings and recalled rating of one illustrative participant over a single day with added mathematical mean value and labels for values of special interest (peak and end). Values were calculated similarly over the 7‐day study period

**FIGURE 3:**
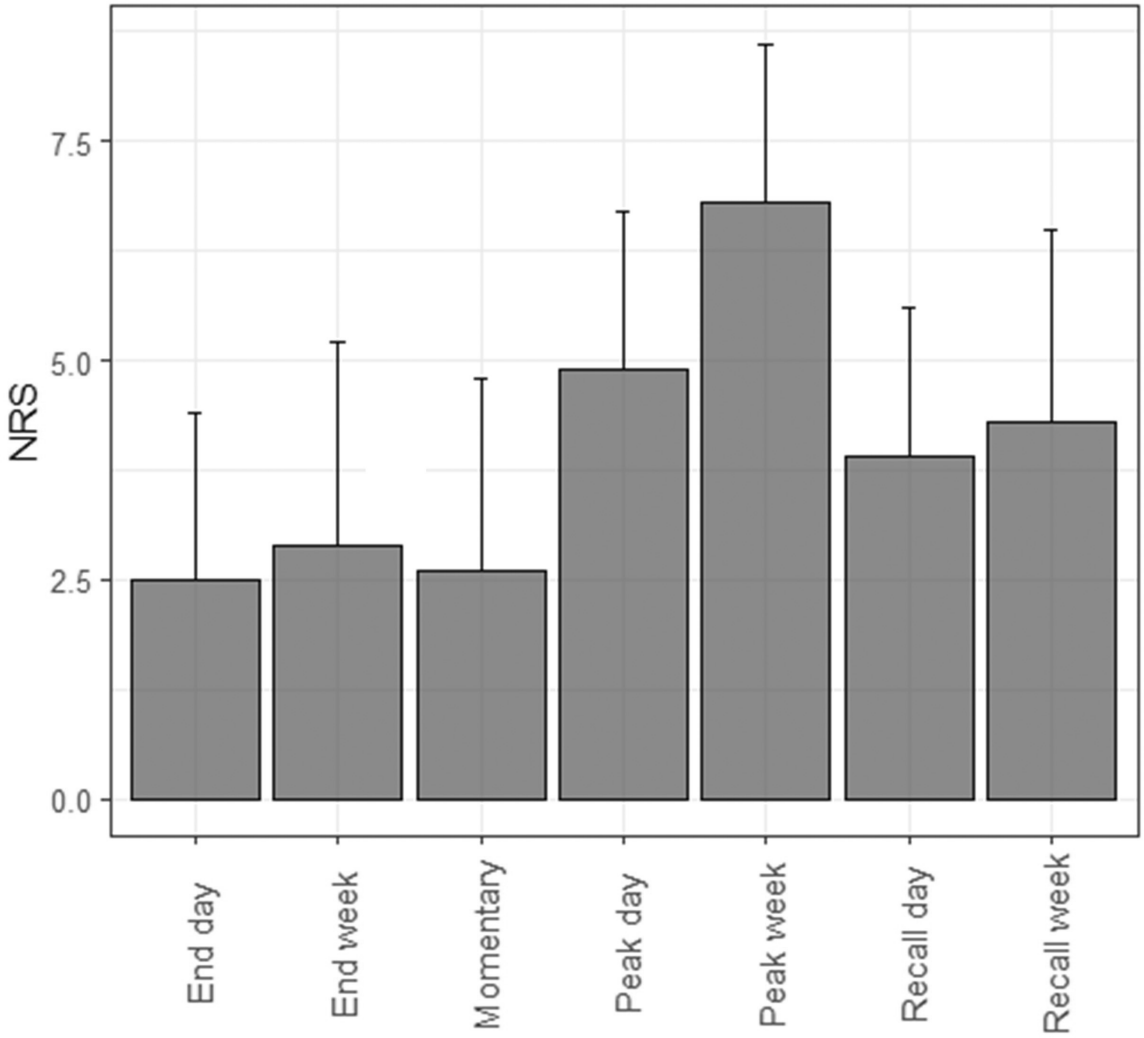
Mean numerical rating scale (NRS) values of momentary breathlessness severity of the entire cohort throughout the 7‐day study

The ratings from one illustrative participant are presented in Figure 2. The mean value of momentary breathlessness ratings throughout the day for the study period was 2.6 (SD 2.2) on the 0–10 NRS, the mean *daily peak value* was 4.8 (1.8) and the mean *weekly peak value* was 6.8 (SD 1.8). The mean *daily recalled value* was 3.9 (SD 1.7), and the mean *weekly recalled value* was 4.3 (SD 2.2; Figure 3).

### Analysis of daily ratings

We observed a significant association between momentary and recalled breathlessness in univariate analyses (Table 2). For each unit increase in an individual rating of momentary breathlessness, the recalled rate for *the day* increased by 0.10 (95% CI 0.08–0.11) units. The mean of the ratings for the day showed the strongest association with the recalled severity for that day, with each unit increase of the mean resulting in a 0.67 (95% CI 0.63–0.71) unit increase in recalled severity. The *recalled value* showed an association with the *peak value* (beta value 0.28 [95% CI 0.26–0.30]). The end value was also positively associated with the recalled value but to a lesser degree. Multivariate analysis with peak and end was similar to univariate findings. Change in momentary breathlessness from the group mean showed no association with recalled breathlessness (Table 2).

**TABLE 2:** Relationship between recalled breathlessness at the end of the day and momentary breathlessness ratings during that day (beta coefficients and corresponding 95% CI)

Factor	Univariate analysis	Multivariate analysis
	Beta (95% CI)	Beta (95% CI)
Momentary	0.097 (0.08–0.11)	‐
Change	0.00 (−0.02–0.02)	‐
Mean	0.67 (0.63–0.71)	‐
Peak	0.28 (0.26–0.30)	0.26 (0.2–0.28)
End	0.16 (0.14–0.18)	0.10 (0.08–0.12)

*Note*: Estimates were analysed using mixed linear regression with random intercepts and slopes, accounting for repeated measurements. *n* = 84.

Abbreviations: Change, difference in breathlessness from the mean value, calculated by subtracting each reported value from the individual mean for that day; end, last recorded breathlessness rating before recall; NRS, numerical rating scale; peak, highest recorded momentary breathlessness rating.

### Analysis of the ratings over the week

Associations between momentary and recalled breathlessness for *the week* are shown in Table 3. Significant associations were revealed for the *peak value* (beta = 0.97, *r* ^2^ = 0.56, *p* < 0.000), the *mean momentary breathlessness* (beta = 0.91, *r* ^2^ = 0.52, *p* < 0.000) and the *end value* (beta = 0.69, *r* ^2^ = 0.50, *p* < 0.000). The relationship was strongest with the *peak value*. The *mean*, *peak* and *end values* were combined pairwise in multivariate models 1–3 and then combined all together for model 4 analysis (Table 3). The peak value consistently made the highest contribution to the models. The association between *mean* and *recalled values* was reduced when combined in the model with the *peak* and *end values*. The unique contribution (Δ*r* ^2^) of the *mean* to model 4 was close to zero (Δ*r* ^2^ = 0.00) and Δ*r* ^2^ = 0.11 for the *peak value* (Table 3).

**TABLE 3:** Relationship between the recalled breathlessness at the end of the week and the momentary breathlessness ratings during that week (beta coefficients, corresponding 95% CI, *r* ^2^ and Δ*r* ^2^)

	Univariate	Model 1	Model 2	Model 3	Model 4
Factor	Beta (95% CI)	Beta (95% CI)	Beta (95% CI)	Beta (95% CI)	Beta (95% CI)
*R* ^2^ for the whole model	‐	*r* ^2^ = 0.66	*r* ^2^ = 0.55	*r* ^2^ = 0.64	*r* ^2^ = 0.66
Mean	0.91 (0.71 to 1.1), *r* ^2^ = 0.52	‐	0.54 (0.20 to 0.89), Δ*r* ^2^ = 0.05	0.49 (0.25 to 0.72), Δ*r* ^2^ = 0.08	0.22 (−0.1 to 0.55), Δ*r* ^2^ = 0.00
Peak	0.97 (0.77 to 1.16), *r* ^2^ = 0.56	0.65 (0.43 to 0.87), Δ*r* ^2^ = 0.16	‐	0.62 (0.38 to 0.87), Δ*r* ^2^ = 0.12	0.59 (0.35 to 0.83), Δ*r* ^2^ = 0.11
End	0.69 (0.53 to 0.85), *r* ^2^ = 0.50	0.38 (0.22 to 0.55), Δ*r* ^2^ = 0.1	0.34 (0.07 to 0.61), Δ*r* ^2^ = 0.03	‐	0.27 (0.04 to 0.50), Δ*r* ^2^ = 0.02

*Note*: Variables were analysed separately (univariate) and together in different combinations (models 1–4). Estimates were analysed using linear regression, *N* = 76.

Abbreviations: Δ*r* ^2^, contribution from each factor to the variance of the model (*r* ^2^ for the whole model − *r* ^2^ for the model without the current factor). A higher Δ*r* ^2^ corresponds to a higher contribution to that model; end, last recorded value of momentary breathlessness before recall; mean, mean value of momentary breathlessness ratings for the week; NRS, numerical rating scale; peak, highest recorded momentary breathlessness rating; *r* ^2^, percentage of the variance explained by the whole model.

Findings were similar when adjusting the associations for age and sex.

## DISCUSSION

The main finding of the study was that the peak momentary breathlessness over the course of 7 days was closely linked with the recalled breathlessness severity for that same period. The findings suggest that the impact of peak breathlessness on recalled breathlessness is stronger than the impact from the *mean* or the *end values* for 1 week. Recall for 1 day seemed to be influenced the most by the *mean breathlessness value* for that day.

This study contributes novel information on interpretation of self‐reported breathlessness levels over 1 day or 1 week. Compared to other studies with a similar methodology, we have collected many more breathlessness ratings and used verifiable real‐time measures.^39^, ^40^, ^49^

Similar to our results, recall of breathlessness after an exercise test has been shown to reflect the impact of peak breathlessness but not the last recorded value.^33^ In our study, we found that the impact of the peak value was stronger when using a 7‐day recall period compared to a daily recall (where the mean value had the strongest association). This could be explained by basic memory functions, suggesting that a shorter recall period decreases bias.^15^, ^34^ No association was found between recall and change in breathlessness (Table 2). This indicates that hourly changes in breathlessness do not impact the recall for that day to any large degree.

A change of 1 point in the *peak* or the *mean value* influenced the weekly recall by a margin of close to 1 point. This corresponds to a large clinically important change as pre‐defined in the protocol.^41^ A change in mean breathlessness of 1 point for the day influenced the recall with an increase of 0.67 points, which corresponds to a moderate change.

Strengths of this study include the use of mEMA as a novel tool to investigate this research question using data captured in real time. The use of mEMA also gives better compliance than paper diaries^35^ and prevents participants from manually changing or adding responses afterwards.^37^ This is the first study of its kind and adds new knowledge based on reliable and consistent use of a 0–10 NRS.

Limitations include the lack of data concerning activities performed when reporting breathlessness, limiting in‐depth interpretation. Our choice to include participants with breathlessness with different aetiology may limit generalizability in disease‐specific groups but, at the same time, improves generalizability among unselected populations. The size of the study population limits the possible subgroup analysis of differences between disease groups. Future studies on more selected populations are needed.

This study was conducted in Swedish, and confirmatory studies using other languages are needed. Selection bias due to participants with mobile phones being younger or healthier could be an issue, but mobile use disparities between generations have decreased substantially in recent years for 65–75‐year‐olds.^36^, ^50^

The study suggests that *peak breathlessness* has an impact on recalled breathlessness. This might be an important consideration in clinical practice where it is often necessary to collect information covering more extended periods, especially in outpatient care. Future research should focus on the clinical relevance of these findings and the relationships with treatment outcomes and survival. For example, would a treatment that reduced the peak breathlessness be more important than one lowering the mean?

In conclusion, recalled severity of breathlessness over the past 7 days was more strongly linked to the *peak momentary breathlessness* in that period than to average or most recent (end) values. Recall for 1 day was influenced the most by the *mean breathlessness value* for that day.

## AUTHOR CONTRIBUTION

**Jacob Sandberg:** Conceptualization (lead); data curation (lead); formal analysis (lead); funding acquisition (lead); investigation (lead); methodology (lead); project administration (lead); resources (lead); software (lead); validation (equal); visualization (lead); writing – original draft (lead); writing – review and editing (equal). **Josefin Sundh:** Conceptualization (supporting); formal analysis (supporting); methodology (supporting); supervision (supporting); writing – review and editing (equal). **Peter Anderberg:** Conceptualization (supporting); formal analysis (supporting); methodology (supporting); software (supporting); writing – review and editing (equal). **David C. Currow:** Conceptualization (supporting); formal analysis (supporting); methodology (supporting); writing – review and editing (equal). **Miriam Johnson:** Conceptualization (supporting); formal analysis (supporting); methodology (supporting); writing – review and editing (equal). **Robert Lansing:** Conceptualization (supporting); formal analysis (supporting); methodology (supporting); writing – review and editing (equal). **Magnus Ekström:** Conceptualization (lead); formal analysis (supporting); funding acquisition (supporting); investigation (supporting); methodology (lead); project administration (supporting); resources (equal); supervision (lead); validation (equal); visualization (supporting); writing – review and editing (lead).

## CONFLICT OF INTEREST

None declared.

## HUMAN ETHICS APPROVAL DECLARATION

The Regional Ethical Review Board in Lund approved the study procedures (DNr 2017/149). All participants provided written informed consent prior to enrolment. Participation could be discontinued at the discretion of the participant, in which case, no further data were collected. All research was performed in accordance with relevant guidelines and regulations.

## Supporting information


**Visual Abstract** Comparing recalled versus experienced symptoms of breathlessness ratings: An ecological assessment study using mobile phone technology

## Data Availability

The data that support the findings of this study are available on request from the corresponding author. The data are not publicly available due to privacy or ethical restrictions.
