# Supplementary material for: Comparing recalled versus experienced symptoms of breathlessness ratings: An ecological assessment study using mobile phone technology
Source: Respirology. 2022 Jun 13;27(10):874–81. doi: 10.1111/resp.14313 (PMC9546302; doi:10.1111/resp.14313)
Supplement: Supplementary file 1 — Visual Abstract Comparing recalled versus experienced symptoms of breathlessness ratings: An ecological assessment study using mobile phone technology [file RESP-27-874-s001.pdf]

Comparing recalled versus experienced symptoms of breathlessness ratings

An ecological assessment study using mobile phone technology

Method

An ecological assessment study using mobile phone technology

Repeated measures each wake hour for one week

Recall each night and at end of week

Results

Study participant number **84**

6152 measurements (7.7/participant/day)

Mean momentary breathlessness was 2.6 (SD 2.2) on the 0–10 NRS

Conclusion

For one day: Strongest influence on recall from the mean value $\bar{X}$

For one week: Strongest influence on recall from the peak value

Respirology

Comparing recalled versus experienced symptoms of breathlessness ratings: An ecological assessment study using mobile phone technology

Sandberg et al. Respirology 2022; DOI: 10.1111/resp.14313
